# Comprehensive Assessment of the Genotype-Environment Interaction and Yield Stability of Boro Rice Genotypes under Four Environments in Bangladesh Using AMMI Analysis

**DOI:** 10.1155/2024/7800747

**Published:** 2024-07-04

**Authors:** Shams Shaila Islam, Md. Borhan Uddin Sarker, Md. Masud Rana, Ahmed Khairul Hasan, Md. Rashed Karim, Thanet Khomphet

**Affiliations:** ^1^ Department of Agronomy Faculty of Agriculture Hajee Mohammad Danesh Science and Technology University, Dinajpur 5200, Bangladesh; ^2^ Department of Agronomy Faculty of Agriculture Bangladesh Agricultural University, Mymensingh 2202, Bangladesh; ^3^ Department of Geography and Environment New Government Degree College, Rajshahi 6000, Bangladesh; ^4^ School of Agricultural Technology and Food Industry Walailak University, Nakhon Si Thammarat 80160, Thailand

## Abstract

Yield stability, alongside high yield potential and broad adaptation to various agroclimatic environments, is a key objective for rice breeders aiming to ensure food security. This study aimed to explore the most suitable and stable Boro rice genotypes for Bangladesh. Ten Boro rice genotypes underwent testing in four environments during the 2022 Boro season to investigate genotype-environment interaction (GEI) and yield stability performance. The experiment utilized three replications of a completely randomized block design. Yield stability performance was assessed through combined analysis and the additive main effects and multiplicative interaction (AMMI) model. The combined ANOVA revealed that the environment explained 10.23%, while GEI accounted for 9.17%, and the genotypes captured 80.60% of the variance, significantly impacting grain yield. Significance was observed in the environment, genotype main effects, and GEI. Analysis indicated that BRRI dhan 68 yielded the highest (6,754 kg·ha^−1^) and BRRI dhan 88 the lowest (5,620 kg·ha^−1^) among the investigated genotypes. In addition, genotypes BRRI dhan 84, BRRI dhan 81, and BRRI dhan 67 exhibited the highest grain yields. The Rangpur environment demonstrated considerable stability across the four environments with a high mean value of grain yield (7,206 kg·ha^−1^). Therefore, the AMMI model emerges as a valuable tool for identifying the most suitable and stable Boro rice genotypes with high-yielding potential across various regions in Bangladesh, as well as under diverse conditions.

## 1. Introduction

Rice (*Oryza sativa* L.) is a staple diet for most of the world's population, providing about 20% of the world's dietary energy needs as valuable cereal [1, 2]. Bangladesh has made tremendous advancements in the production of rice over the past 30 years and is regarded as a nation of rice cultivators and consumers in the world [3]. According to research studies, the intensification of social, economic, and demographic environmental pressures will demand an additional 112 million metric tons of rice to meet global human intake by 2035 [4]. In addition, Bangladesh's population is growing by two million each year and might add 30 million more people in the next 20 years. As the population forecast for Bangladesh is for an increase from the current 169 million to 220 million by 2050, the importance of rice production becomes even clearer. In recent years, however, overall profitability has declined due to rising input prices and increasing labour costs, which make rice cultivation more difficult [3]. Reducing income volatility and increasing profitability is an important step towards increasing social welfare and the sustainability of rice production [5]. Yield is closely linked to profitability, and increases in yield have helped rice farming to remain profitable, especially after 2005 in Bangladesh [6]. Approximately 0.15% of the country's arable land is diverted from agriculture each year due to the demand for additional dwellings and business enterprises to meet the expanding population, and Bangladesh currently faces a tremendous problem in preserving food self-sufficiency. To supply the growing demand for food in Bangladesh, it is crucial to utilize genotypic adaptability, current agricultural techniques, and water management [7]. Therefore, genotypic adaptability to environmental change is critical for crop yield stability across locations and years. Furthermore, high yielding genotypes are critical to the food security program because they improve output potential and yield stability [8].

The three seasons of Aus, Aman, and Boro are used to cultivate rice throughout the year in Bangladesh. Boro rice is one of the principle types and accounts for a substantial portion of the nation's overall rice production [9]. However, Bangladesh has a great subtropical environment for Boro rice productivity, which is low in comparison to other Asian countries, e.g., Indonesia and Malaysia. According to the Bangladesh Bureau of Statistics [10], the target for cultivating Boro rice is set at 4,872,600 hectares for the fiscal year 2021-2022. Until February 27, farmers had brought 27,07,572 hectares (76.61% of the target) under Boro cultivation. The average Boro rice yield in Bangladesh varies depending on a number of variables, including the rice genotypes, the region, the season, inefficient nutrient management, disease, stresses such as drought, flood, salinity, extreme temperature, low soil fertility, and agricultural practices used, all of which reduce rice yields and increase their variability as well as yield stability in Bangladesh [11]. Therefore, extensive Boro rice cultivation is being made possible by improving crop management, increasing the application of appropriate fertilizer, pest management, and yield stability over a genotype of environments.

Yield stability is one of the primary objectives of improving genotypes with high potential for yield along with improved and broad adaptation to varied agroclimatic environments and it is the goal of rice breeders in a food security package because multilocation genotype evaluation enables plant breeders to assess a genotype's adaptation to a given environment as well as its stability over a range of habitats [12]. A genotype's level of interaction with various growing conditions is usually utilized to assess how well it has adapted to various environments. As a result, a genotype is considered more adaptable or stable if it has a high mean yield with low variation for yield capability when grown in different environments or locations. However, in order to generate adaptive and high yield genotypes, an environmental evaluation must be conducted because GEI may affect yield and other performance variation [13]. Furthermore, when making decisions about rice cultivation, farmers always consider the expected yields against production costs. However, due to fluctuations in the market price of rice [6], volatility in rice income has become the norm among rice farmers in Bangladesh [14].

For effective policy interventions, it is crucial for the government to analyze the cost, actual yield, profitability, and risk associated with rice cultivation under various growing conditions, such as different locations, varieties, input usage, irrigation sources, and planting dates. Consequently, to measure the stability and yield performance of rice genotypes, site-specific genotypes must be selected through extensive environmental experiments. Multienvironment trials, based on yield performance and genotype stability across locations, can identify superior genotypes and offer valuable guidance for breeders in selecting optimal genotypes [15]. Thus, this study aimed to explore the most suitable and stable Boro rice genotypes for Bangladesh by assessing in four environments using yield stability and AMMI model.

## 2. Materials and Methods

### 2.1. Planting Material

**Figure 1:**
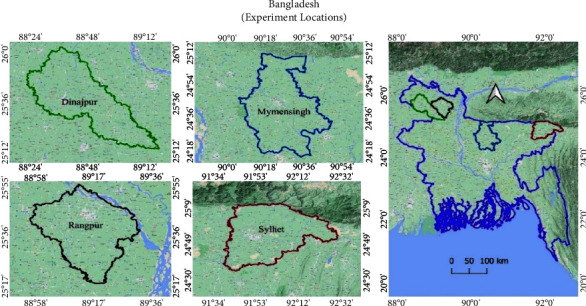
The geographical experimental four different locations in Bangladesh.

Four environments, namely, Dinajpur (E_1_), Rangpur (E_2_), Mymensing (E_3_), and Sylhet (E_4_), were utilized for the investigation between January 2022 and April 2022 to assess Boro rice genotypes for yield-contributing traits over a single year. Geographic details of the four experimental locations, including their altitudes, latitudes, and longitudes, are depicted in Figure 1. This study involved 10 rice genotypes developed at the Bangladesh Rice Research Institute (BRRI), Gazipur, with genotype being the primary determinant and environments serving as subfactors. The experimental design included three replications following a completely randomized block design. Each plot measured 4 meters by 2.5 meters, with a 1-meter gap between replications. The spacing between each line was 30 cm. Seeds were sown in nursery plots on January 14, 2022, and after 35 days, the seedlings were transplanted to the main field on February 17, 2022.

The experimental field received fertilization with 10 tons of cowdung, 170 kg of triple superphosphate (TSP), 250 kg of urea, 85 kg of muriate of potash (MoP), 150 kg of gypsum, and 10 kg of boric acid per hectare, following the fertilizer recommendation guide of 2018 in Bangladesh. During the final stage of land preparation, all fertilizers except urea, MoP, TSP, zinc oxide, gypsum, and boron were applied. In addition, urea was divided into three equal doses: one during the final land preparation phase, one-half during the vegetative stage, and the remaining half at maturity. Consistent weeding, thinning, irrigation, pest treatment, and other intercultural practices were implemented in all plots. Throughout the growth season, an effective drainage system was maintained to ensure the prompt flow of rainfall from the experimental plot. The first and second weedings were completed 15 and 35 days after planting, respectively. Simultaneously, thinning was performed to maintain a gap of 10 cm between plants. During the vegetative stage, pests such as rice bugs and green plant hoppers were observed in the crop, and chlorpyrifos 20% EC was applied at a concentration of 2 ml per liter of water to control the infestation.

### 2.2. Test Environment with Soil Characteristics

In the multienvironment experiment, the study was conducted across four distinct environments, and genotypes were evaluated for one season in four locations, accounting for variations in crop quantity and quality due to factors such as animal presence, pests, and diseases. Differentiating factors in the study included soil type, site conditions, planting time, and season. Genotypic responses were assessed based on parameters such as temperature, relative humidity (RH), sunlight exposure, and rainfall. Each combination of planting site and season was considered a unique environment for evaluating genotypic responses. The soil properties for each environment are detailed in Table 1.

### 2.3. Climatic Condition

**Figure 2:**
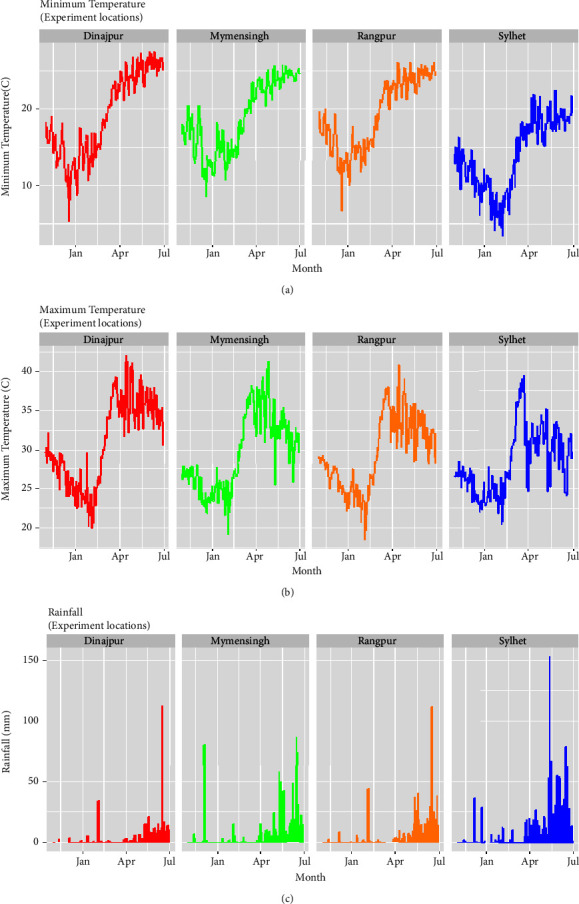
Maximum (a) and minimum (b) temperature, and rainfall (c) ranges for four environments in Bangladesh.

With notable variations in climate factors such as temperature and rainfall, Bangladesh exhibits a tropical climate. Four selected locations were analyzed by measuring maximum and minimum temperatures, as well as rainfall (Figure 2(a)). These weather data were obtained from the Bangladesh Meteorological Department. Results showed that the maximum temperature was recorded as 44°C in E_1_ (Dinajpur) during April to May, 41°C in E_2_ (Rangpur) in late May, 42°C in E_3_ (Mymensing) in April, and 39°C in E_4_ (Sylhet) in late March. Based on these maximum temperatures, the environments experienced high temperatures ranging from 28°C to 44°C, from E_1_ (Dinajpur) to E_4_ (Sylhet). Similarly, minimum temperatures were recorded as 28°C in E_1_ (Dinajpur), 26°C in E_2_ (Rangpur), 26°C in E_3_ (Mymensing), and 22°C in E_4_ (Sylhet). All environments exhibited their highest minimum temperatures in late June to July, with mean minimum temperatures ranging from 22°C to 28°C across E_1_ (Dinajpur) to E_4_ (Sylhet) (Figure 2(b)). The coldest months were January and February, with daily average temperatures ranging from 10°C to 20°C across all environments. Despite variability across locations, Bangladesh receives abundant rainfall, particularly during the monsoon months of June and July. Thunderstorms and premonsoon rain occur in April and May. In E_1_ (Dinajpur), the highest rainfall was recorded in July with 115 mm, followed by 85 mm in E_2_ (Rangpur), 112 mm in E_3_ (Mymensing), and 155 mm in E_4_ (Sylhet) in June. Consequently, the maximum rainfall ranges between environments were observed to be 85 mm to 155 mm, from E_2_ (Rangpur) to E_4_ (Sylhet) (Figure 2(c)).

### 2.4. Statistical Analysis

#### 2.4.1. Analysis of Variance

In the initial stages, a pooled analysis of variance was performed on the data.  Table 2 provides the ANOVA's structure.

(1) $$\begin{array}{c} \text{Genotypic variance }\left(\sigma_{g}^{2}\right)=\frac{\text{ MS}1-\text{MS}2}{r},\\ {\text{Phenotypic variance }\left(\sigma_{P}^{2}\right)=\sigma }_{g}^{2}+\sigma_{e}^{2}, \end{array}$$

where *r* = number of replications, s = number of sowing dates (environment), *v* = number of genotypes, *σ*_*g*_^2^ = genotypic variance, and *σ*_*P*_^2^ = phenotypic variance

#### 2.4.2. Heritability

The ratio of genotypic variance to total variance, or phenotypic variance, was used to determine the broad-sense heritability (*H*_*b*_^2^). The formula (2), [16] is given below:

(2) $$\begin{array}{c} H_{b}^{2}=\frac{\sigma_{g}^{2}}{\sigma_{P}^{2}}\times 100, \end{array}$$

where *σ*_*g*_^2^ and *σ*_*P*_^2^ are the genotypic and phenotypic variance.

#### 2.4.3. Genetic Advance and Genetic Advance as a Percentage of Mean

Following the equation given by Johnson et al. [16], genetic advance (GA) (equation (3)) was calculated for each character and represented as a percentage of mean (GAM) (equation (4)).

(3) $$\begin{array}{c} GA=k\times \sigma_{p}\times h^{2}, \end{array}$$

(4) $$\begin{array}{c} \text{GAM}=\frac{\text{Genetic advance}}{\overset{¯}{X}}\times 100, \end{array}$$

where *h*^2^ = heritability (board sense), *σ*_P_ = phenotypic standard deviation, *k* = standardized selection differential (*k* = 2.06), and $\overset{¯}{X}$ = overall mean of the character concerned.

#### 2.4.4. Stability Analysis

According to Eberhart and Russell [17], the stability analysis (5)was completed. The fundamental model used is as follows:

(5) $$\begin{array}{c} Y_{ij}={µ}_{i}+\beta_{i}I_{j}+\sigma_{ij}, \end{array}$$

where *Y*_*ij*_ = mean of the *i*^th^ genotype at *j*^th^ environment, *μ*_*i*_ = mean of the *i*^th^ genotype over the environments, *β*_*i*_ = regression coefficient of *i*^th^ genotype to varying environmental indices, *I*_*i*_ = environmental index, *i.e.,* mean of all genotypes at *j*^th^ environment minus grand mean, and *σ*_*ij*_ = deviation from regression of *i*^th^ genotype at *j*^th^ environment.

#### 2.4.5. Joint Regression Analysis

The formula for calculating the sum of squares for each source using joint regression analysis is presented in Table 3. The impact of genotypes, environments, genotypes environments interaction, environment + (genotypes × environments) interaction environment (linear), and genotypes × environments (linear) on variance was assessed in comparison to pooled error. However, in order to test the pooled deviation (equation (6)), the pooled error was compared to the pooled deviation using the following formula:

(6) $$\begin{array}{c} \text{MS}=\frac{\text{Pooled error}}{r}, \end{array}$$

where *r* = number of replications.

#### 2.4.6. Stability Parameters

According to Eberhart and Russell [17], stability of rice genotypes was assessed based on the regression coefficient (*b*_*i*_) (equation (7)) and deviation from regression (*S*_*d*_^2^) (equation (8)).

(7) $$\begin{array}{c} \text{Regression coefficient }\left(b_{i}\right)=\frac{\sum_{j}Y_{\mathrm{ij}}I_{j}}{\sum_{j}I_{j}^{2}}, \end{array}$$

(8) $$\begin{array}{c} S_{d}^{2}=\frac{E_{j}\sigma {\text{ij}}^{2}}{s-2}-\frac{Se^{2}}{r}, \end{array}$$

where ∑_*j*_*σ*^2^*ij*=(∑*jY*_*ij* _^2^ − *Yi*^2^/*S*) − ∑_*j*_*Y*_*ij*_*Ij*^2^/∑*jIj*^2^.

(9) $$\begin{array}{c} \left(\sum jY_{\mathrm{ij}\;}^{2}-{\mathrm{Yi}}^{2}/S\right)=\text{variance due to dependent variable}, \end{array}$$

where ∑_*j*_*Y*_*ij*_*Ij*^2^/∑*jIj*^2^ = variance due to regression.

A stable genotype is one that has a deviation (*S*_*d*_^2^) that is equal to zero and a regression coefficient (*b*_*i*_) that is equal to one (*b*_*i*_ = 1.0). These stability criteria, along with the mean value of the attributes, are used to determine how desirable a genotype is. The regression coefficient (*b*_*i*_) (equation (10)) was obtained as follows:

(10) $$\begin{array}{c} \text{Standard error of regression coefficient }\left(b_{i}\right)={\left(\frac{\text{MS due to pooled deviation due to }i^{\text{th}}\text{genotype }}{\sum {\text{jI}}_{j}^{2}}\right)}^{1/2}. \end{array}$$

## 3. Results

### 3.1. Combined Analysis of Variance

The combined analysis of pooled variance (Table 4) revealed highly significant differences among the genotypes for most characters. However, panicle length (22.25 cm) and total grain weight (18.22 g) showed a nonsignificant relationship across all environments. In addition, the pooled analysis of variance indicated significant differences among environments for all characters. The mean square due to the environment was highly significant for each character, including plant height (445.72), tiller number per plant (64.20), panicle length (81.04), panicle number per plant (393.79), total dry weight (106.55), total grain weight (39.06), 1,000-seed weight (2,578.14), harvest index (15,229.20), leaf area index (0.96), filled grain per plant (19,389.10), unfilled grain per plant (6,724.50), and grain yield (18,570.59).

Furthermore, the genotype-environment interaction (GEI) was highly significant for plant height (98.22), panicle number per plant (7.35), tiller number per plant (4.70), total grain weight (315.75), harvest index (11.60), unfilled grain per plant (3,775.0), filled grain per plant (15,532), and grain yield (791,247). However, panicle length (10.58), total dry matter (20.94), 1,000-seed weight (9.20), and leaf area index (0.24) showed a nonsignificant relationship with GEI.

#### 3.1.1. Mean Performance Comparison of Grain Yield over Different Environments

The comparison of mean grain yield performance among the ten Boro rice genotypes is presented in Table 5. Among all the genotypes, G_5_ and G_6_ exhibited the highest grain yields, with values of 8405 kg·ha^−1^ and 6753 kg·ha^−1^, respectively. In addition, G_7_ (6,705 kg·ha^−1^), G_8_ (6,659 kg·ha^−1^), and G_10_ (6,072 kg·ha^−1^) demonstrated moderate performance. Conversely, G_9_ (5,619 kg·ha^−1^) and G_1_ (5,800 kg·ha^−1^) were the poorest yielders, with the lowest grain yield recorded for G_9_ (5,619 kg·ha^−1^).

On an overall mean basis, genotype G_8_ yielded the highest at E_1_ (Dinajpur) with 7,744 kg·ha^−1^, while genotype G_5_ achieved the highest yield at E_2_ (Rangpur) with 8,797 kg·ha^−1^. Genotype G_7_ performed the best at E_3_ (Mymensing) with 6,344 kg·ha^−1^, and at E_4_ (Sylhet), genotype G_6_ had the highest yield at 6,241 kg·ha^−1^. Conversely, genotypes G_1_, G_9_, G_3_, and G_6_ consistently exhibited the lowest yield across different environments, with yields of 5,535 kg·ha^−1^ at E_1_ (Dinajpur), 6,038 kg·ha^−1^ at E_2_ (Rangpur), 5,238 kg·ha^−1^ at E_3_ (Mymensing), and 4,953 kg·ha^−1^ at E_4_ (Sylhet). Consequently, genotype G_5_ emerged as the best performer across the various environments.

### 3.2. Range and Mean Variance Result of Ten Boro Rice Genotypes

In the present investigation (Table 6), a comparison of the means across genotypes for various traits in four different environments revealed that E_1_ (Dinajpur) exhibited the highest mean for panicle number per plant (17 no.), harvest index (45.95%), and the lowest mean for plant height (113.50 cm), tiller number per plant (8 no.), leaf area index (1.68%), and unfilled grain per plant (240 no.). E_2_ (Rangpur) displayed the highest mean for leaf area index (2.10%), filled grain per plant (790 no.), and grain yield (7,205 kg·ha^−1^), while E_3_ (Mymensing) showed the highest mean for unfilled grain per plant (275 no.) and the lowest for filled grain per plant (630 no.). The mean values for plant height, total dry weight per plant, total grain weight per plant, and 1,000-seed weight were 122.00 cm, 43.14 g, 27.33 g, and 43 g, respectively.

Furthermore, the results indicated that only the harvest index exhibited a better mean in the E_1_ (Dinajpur) environment, whereas E_2_ (Rangpur) and E_4_ (Sylhet) exhibited superior mean values for leaf area index, filled grain per plant, grain yield, total dry weight per plant, total grain weight per plant, and 1,000-seed weight. Comparison of ranges (Table 6) across different traits in the four environments revealed that E_4_ (Sylhet) exhibited the highest range for plant height (117–124 cm), total dry weight (38–45 g), total grain weight (22.95-30.46 g), 1,000-seed weight (40–46 g), and harvest index (55–62). On the other hand, E_1_ (Dinajpur) had the widest range only for panicle number per plant (15–21 no.). The second widest range was observed in E_2_ (Rangpur) for panicle length (24.40–29.0 cm), leaf area index (1.71–2.39%), filled grain per plant (696–904 no.), and grain yield (6,038−8,797 kg ha^−1^). E_3_ (Mymensing) exhibited the widest range for tiller number per plant (14–16 no.) and unfilled grain per plant (214–368 no.). The results highlighted the flexibility of all environments in terms of yield-contributing traits, with E_4_ (Sylhet) and E_2_ (Rangpur) showing the widest ranges for most of these traits, thus making them ideal environments for screening genotypes.

### 3.3. Variance for Genotype and Phenotype

Variance for genotype (*σ*_*g*_^2^) and phenotype (*σ*_*p*_^2^) are categorized into low, moderate, and high [18]. Results from the present study (Table 7) revealed that in E_1_ (Dinajpur), high genotypic variance (*σ*_*g*_^2^) and phenotypic variance (*σ*_*p*_^2^) were observed for plant height (14.75% and 23.56%), panicle number per plant (13.99% and 28.72%), harvest index (7.42% and 22.00%), and leaf area index (24.11% and 58.67%). Moderate values were recorded for filled grain per plant (11.11% and 21.00%), unfilled grain per plant (11.66% and 21.63%), and grain yield (5,854 and 12,224.11%). Low values were noted for tiller number per plant (3.77% and 13.56%), panicle length (2.89% and 6.59%), total dry weight (5.67% and 8.75%), and total grain weight (5.45% and 7.49%).

In E_2_ (Rangpur), high values were observed for panicle number per plant (12.99% and 23.12%), leaf area index (17.25% and 35.00%), and grain yield (6773 and 12445%). Moderate values were found for total dry weight (10.56% and 8.77%), total grain weight (11.86% and 16.23%), filled grain (11.13% and 25.34%), and unfilled grain (12.00% and 22.13%). Low values were recorded for plant height (3.77% and 13.56%), panicle length (2.89% and 6.59%), total dry weight (9.54% and 17.56%), tiller number (4.77% and 8.88%), 1,000-seed weight (6.19% and 12.12%), and harvest index (4.11% and 7.67%).

In E_3_ (Mymensing), high values were noted for panicle number per plant (14.71% and 23.11%), leaf area index (17.25% and 35.00%), and grain yield (6773 and 12445%). Moderate values were recorded for filled grain (12.00% and 18.34%) and unfilled grain (10.35% and 21.40%). Low values were observed for tiller number (4.71% and 7.78%), panicle length (5.13% and 8.40%), total grain weight (9.54% and 17.56%), tiller number (5.00% and 6.77%), 1,000-seed weight (6.39% and 12.00%), and harvest index (6.52% and 13.44%).

In E_4_ (Sylhet), high values were recorded for plant height (13.40% and 18.20%), panicle number per plant (13.17% and 16.34%), leaf area index (24.00% and 28.10%), and grain yield (5,554 and 12,446%). Moderate values were observed for filled grain (10.00% and 23.30%) and unfilled grain (11.15% and 23.44%). Low values were noted for tiller number (3.40% and 6.77%), panicle length (4.67% and 8.90%), total dry weight (5.71% and 9.12%), total grain weight (5.10% and 7.89%), 1,000-seed weight (4.03% and 9.30%), and harvest index (4.14% and 9.34%).

### 3.4. Genotypic and Phenotypic Coefficient of Variance

Results from the present study (Table 8) indicate that in E_1_ (Dinajpur), there were high genotypic and phenotypic coefficients of variance (GCV and PCV) for tiller number per plant (13.48% and 26.08%), harvest index (16.15% and 33.88%), filled grain per plant (16.90% and 17.16%), and grain yield (14.23% and 31.77%). Moderate GCV and PCV values were observed for plant height (5.10% and 11.89%), panicle number per plant (4.61% and 13.12%), total dry weight (4.32% and 12.98%), 1,000-seed weight (8.27% and 21.21%), and leaf area index (9.77% and 46.36%). Low GCV and PCV values were recorded for panicle length (2.23% and 4.27%), total grain weight (2.02% and 4.34%), and unfilled grain per plant (2.04% and 6.35%).

In E_2_ (Rangpur), high GCV and PCV values were observed for tiller number per plant (13.90% and 26.46%), harvest index (16.09% and 33.88%), filled grain per plant (15.13% and 15.47%), and grain yield (16.90% and 31.43%). Moderate GCV and PCV values were recorded for plant height (8.14% and 14.50%), panicle number per plant (4.61% and 13.12%), total dry weight (3.44% and 14.04%), 1,000-seed weight (8.56% and 25.58%), and leaf area index (6.59% and 46.28%). Low GCV and PCV values were noted for panicle length (2.98% and 4.89%), total grain weight (2.89% and 4.62%), and unfilled grain per plant (2.04% and 6.35%).

In E_3_ (Mymensing), high GCV and PCV values were observed for tiller number per plant (13.55% and 27.82%), harvest index (15.99% and 23.82%), filled grain per plant (10.00% and 20.55%), and grain yield (13.80% and 33.26%). Moderate GCV and PCV values were recorded for panicle number per plant (8.35% and 15.84%), total grain weight (5.66% and 5.73%), 1,000-seed weight (9.81% and 20.30%), and leaf area index (8.85% and 46.31%). Low GCV and PCV values were observed for plant height (1.28% and 9.05%), total dry weight (1.50% and 12.64%), panicle length (2.80% and 4.24%), and unfilled grain per plant (2.04% and 5.31%).

In E_4_ (Sylhet), high GCV and PCV values were recorded for tiller number per plant (13.90% and 26.46%), harvest index (16.15% and 33.88%), filled grain per plant (10.56% and 24.33%), leaf area index (14.59% and 47.5%), and grain yield (18.41% and 30.13%). Moderate GCV and PCV values were observed for panicle number per plant (4.61% and 13.12%), total dry weight (7.13% and 33.54%), and 1,000-seed weight (10.56% and 24.33%). Low GCV and PCV values were noted for plant height (2.67% and 8.00%), total grain weight (3.40% and 4.12%), panicle length (2.18% and 4.54%), and unfilled grain per plant (2.04% and 6.35%). Therefore, the GCV ranged from 1.28% in E_3_ (Mymensing) to 18.41% in E_4_ (Sylhet), while the PCV ranged from 4.12% to 47.51% obtained from E_4_ (Sylhet).

## 4. Heritability

Broad-sense heritability was categorized into high (>80%), moderate (60–80%), and low (<60%). High heritability values were noted in this study for all the quantitative characters in four environments, ranging from 62.16% to 97.97%. This suggests that the phenotypic expression was largely determined by genotypic differences, with minimal influence from environmental factors (Table 9). The characters with high heritability included plant height (87.23%), tiller number per plant (86.71%), total grain weight (85.21%), panicle number per plant (97.97%), harvest index (82.73%), filled grain per plant (96.19%), and grain yield (88.4%) in E_1_ (Dinajpur); plant height (87.11%), tiller number per plant (87.61%), total grain weight (91.21%), panicle number per plant (97.37%), harvest index (82.55%), filled grain per plant (91.0%), and grain yield (81.5%) in E_2_ (Rangpur). In addition, tiller number per plant (87.61%), panicle length (83.57%), panicle number per plant (95.13%), harvest index (85.06%), filled grain per plant (91.0%), and grain yield (78.5%) were observed in E_3_ (Mymensing), while panicle length (80.33%), harvest index (82.73%), filled grain per plant (88.82%), and grain yield (80.16%) were observed in E_4_ (Sylhet) environment.

### 4.1. Genetic Advance as Percentage of Mean

The GAM was categorized as low (<10%), moderate (10–20%), and high (>20%) [16]. Results revealed high GAM for tiller number per plant (35.05%) in both E_2_ (Rangpur) and E_4_ (Sylhet). The highest panicle number per plant (32.02%) was recorded in E_2_ (Rangpur). Furthermore, high GAM was observed for 1,000-SW (51.35%) in both E_1_ (Dinajpur) and E_2_ (Rangpur), and a substantial percentage of filled grain per plant (37.50% in E_2_ Rangpur, 35.43% in E_4_ (Sylhet)) was noted, while unfilled grain per plant showed high GAM (68.72% in E_2_ Rangpur, 63.17% in E_4_ (Sylhet)).

In this study, moderate GAM was obtained for plant height (23.81%), panicle length (19.06%), and harvest index (22.11%) in E_3_ (Mymensing). Moderate GAM suggests a balanced influence of both additive and nonadditive genes on the control of these traits. In addition, this study reported low GAM for total grain weight (5.90%) and leaf area index (1.92%) in E_2_ (Rangpur).

### 4.2. Joint Regression Analysis of Variance

Joint regression analysis for yield and its components has been performed in following the model provided by Eberhart and Russell [17], and the results are shown in Table 10. In the joint regression analysis, the pooled deviation of the mean sum of square was tested against the pooled error mean sum of square, and where it was significant, all the other mean square were tested against pooled deviation mean sum of square. If pooled deviation mean sum of square was nonsignificant, then pooled error was used for testing the statistically significant of the mean square. The joint regression analysis revealed significant variance due to genotype for all characters. Nonsignificant difference among environments (genotype × environment) was observed for all the characters. Nonsignificant variance due to environment (linear) was also found for all the characters in the experiment. Results showing variance due to genotype × environment (linear) was significant for plant height, tiller number per plant, panicle number per plant, total dry weight, total grain weight, 1,000-seed weight, harvest index, filled grain per plant, unfilled grain per plant, and grain yield, while nonsignificant for panicle length and leaf area index [19].

### 4.3. Stability Analysis

Stability performance of a genotype is judged based on linear regression coefficient (*b*_*i*_) and nonlinear deviation from regression (*S*_*d*_^2^) for yield performance over the environments [2]. According to Finlay and Wilkinson [20], regression coefficient (*b*_*i*_ = 1) indicated average stability, while greater than unity (*b*_*i*_ > 1) indicated below average stability, and less than unity (*b*_*i*_ < 1) indicated above average stability. Deviation from regression (*S*_*d*_^2^) if significant, the performances of a genotype for a given environment may be predicted. In this study, the *b*_*i*_ ranged from −0.02 to 1.79. Genotype G_5_ (BRRI dhan 67) is considered the most stable due to a *b*_*i*_ value closer to unity. Contrarily, genotype G_2_ (BRRI dhan 28) was considered the lowest in rank (Table 11). G_3_ (BRRI dhan 89) and G_8_ (BRRI dhan 84) had a negative linear regression coefficient (*b*_*i*_) for most of the characters and were grown only in poor environments. Besides, G_2_ (BRRI dhan 28), G_5_ (BRRI dhan 67), G_9_ (BRRI dhan 88), and G_10_ (BRRI dhan 29) had below linear regression coefficient (*b*_*i*_) for most of the characters and were more stable over the environments.

### 4.4. AMMI Analysis

From the result of AMMI analysis of variance (Table 12), there are highly significant differences in the environment, genotype, and GEI, *i.e.,* genotype environment interaction (GEI). The combined ANOVA revealed that the environment had a considerable impact on grain yield due to significant variance at the 0.1% level, which accounted for 10.23% of the environment's total variation, while the genotypes and GEI accounted for 9.17% and 80.60%, respectively, of the total sum square. The three main principle components of GEI factors were significant and together accounted for 100% of the overall GEI impact variance in grain yield. A total of 66.5% of the variation produced by the interaction was accounted for by the first principle interaction component (IPCA1), followed by 23.7% (IPCA2) and 9.8% (IPCA3). The first two bilinear terms jointly accounted for 90.2% of the G × E sum of squares and used 20 of the total 27 degrees of freedom available in the interaction. Therefore, the first two IPCA indicated the model has a strong relationship between the characters and fit to describe stability.

### 4.5. IPCA Interactions with AMMI Analysis

The relationships between the IPCA1 and IPCA2 and the mean of genotypes and environments are visually expressed using the AMMI analysis. A multivariate approach called principle component analysis can identify figure arrangements as well as similarities and contrasts between the variables that have been established and arranged in a consecration operation of multivariate systems.  Table 13 displayed the IPCA1 to IPCA3 scores that describe how a genotype interacts with various environments and how genotypes and environments relate to one another. A genotype that scores highly on the IPCA in a number of contexts must counteract negative interactions in other environments [21]. These results demonstrate an uneven genotype response to the environment. Nevertheless, significant major interactions exist and are persistent for both positive and negative indications, as well as genotypes and environments with high IPCA scores. The genotypes that do best in these environments and are stable are those with IPCA1 and IPCA2 scores of zero or almost zero. However, these genotypes interact very little across environments. Conversely, genotypes with negative IPCA1 and IPCA2 values did not interact across environments [22]. As a result, among the ten genotypes, G_6_ (BRRI dhan 68), G_7_ (BRRI dhan 81), G_8_ (BRRI dhan 84), G_2_ (BRRI dhan 28), G_4_ (BRRI dhan 100), and G_10_ (BRRI dhan 29) were generally high yielding with the highest mean values (6,754, 6,705, 6,660, 6,406, 6,341, and 6,180 kg·ha^−1^, respectively) with the environments.

## 5. Interaction of AMMI Model with PCA1 Values

**Figure 3:**
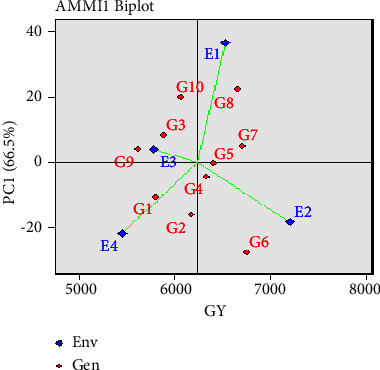
AMMI 1 biplot using IPCA1 and mean grain yield data for ten Boro rice genotypes in four environments.

The IPCA1 and mean of genotypes with environments connection is visually expressed by the AMMI biplot (Figure 3). The interaction pattern was best explained by the first interaction principle component axis (AMMI component 1), which was highly significant. The mean yield was displayed against the IPCA1 scores for the genotypes and the environments, respectively. The relationships between the genotypes and the environments may be easily shown by plotting both the genotypes and the environments on the same graph. In the AMMI analysis, the IPCA scores of genotypes provide a measure of its environmental stability or adaptability. If the IPCA score is a relative number, the higher the score, positive or negative, the more specifically a genotype has adapted to a certain environment. The more closely the IPCA scores approximate zero, the more stable or adaptive the genotype was in the investigated environments as a whole.

The “0” is a perpendicular line in this case. The illustration demonstrates that genotypes with environments on the right side (both higher and lower) of the perpendicular line always have the greatest mean values of grain production. More high mean grain yield values are found in the upper right quadrant than the lower right quadrant, which has medium mean grain yield values. The lowest mean grain yield figures are shown on the left. Using the perpendicular line's performance as a benchmark, genotypes in the E_2_ (Rangpur) environment with a high mean value of grain yield (7,206 kg·ha^−1^) had high mean values and favourable interactions with IPCA1. Genotypes G_2_ (BRRI dhan 28) and G_6_ (BRRI dhan 68) had high mean values with the E_2_ (Rangpur) environment and were stable genotypes with the environment. In addition, genotypes G_8_ (BRRI dhan 84), G_7_ (BRRI dhan 81), and G_5_ (BRRI dhan 67) had high mean values and stability with the E_1_ (Dinajpur) environment. Besides, G_10_ (BRRI dhan 29), G_3_ (BRRI dhan 89), and G_9_ (BRRI dhan 88) had stability in E_3_ (Mymensing) environment. In contrast, E_4_ (Sylhet) is the poorest environment among the four, as shown on the left side of the perpendicular line, and G1 (BRRI dhan 58) had the best-suited genotype for this environment with a yield of 5,801 kg ha^−1^. The E_1_ (Dinajpur) and E_2_ (Rangpur) environments are on the right side of the vertical axis, indicating rich environments, whereas the E_3_ (Mymensing) environment and E_4_ (Sylhet) environment are generally poor environments. Thus, the AMMI biplot shows that the studied genotypes differed from each other not only in their interactive effects but also in their mean grain yield values, as shown in Figure 3.

### 5.1. Interaction Biplot of AMMI Model with PCA1 and PCA2 Values

**Figure 4:**
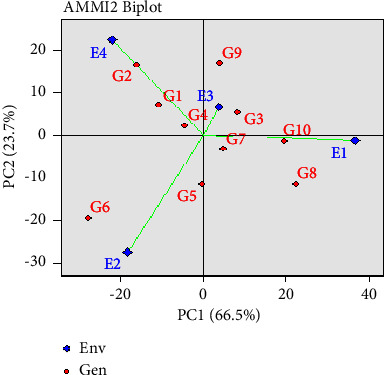
AMMI 2 biplot using IPCA1 and IPCA2 scores data for grain yield with ten Boro rice genotypes in four environments.

The relationships between the mean of genotypes and environments and the IPCA1 and IPCA2 are visually expressed in the AMMI biplot (Figure 4).  Table 13 displays the IPCA1 and IPCA2 scores that describe how a genotype interacts with various settings and how genotypes and environments relate to one another. According to Islam et al. [23], a genotype that scores highly on the IPCA in a number of contexts must neutralize negative interactions in other environments. These results demonstrate an uneven genotype response to the environment. However, both positive and negative signals, as well as genotypes and environments with high IPCA scores, exhibit strong, stable interactions. The genotypes that do best in these contexts and are stable are those with IPCA1 and IPCA2 scores of zero or almost zero. However, these genotypes interact little across environments. There was no interaction between genotypes with negative IPCA1 and IPCA2 values across environments, and all of them had yields which were below average. Similarly, those genotypes have zero scores on the IPCA1, suggesting that the environment has less of an impact on them.

In addition, because they are used to stable conditions and are often adaptable to all environments, the genotypes indicated above typically provide an IPCA1 score close to zero. For grain yield, the biplot showed G_6_ (BRRI dhan 68) had the highest mean value (6,754 kg·ha^−1^) with E_2_ (Rangpur) environment. The maximum mean value was 7,206 kg·ha^−1^, and the interactions were strong. The second highest mean value G_5_ (BRRI dhan 67) with 6,073 kg·ha^−1^ was shown in E_2_ (Rangpur) environment. Among the experiments for grain yields G_1_ (BRRI dhan81) and G_4_ (BRRI dhan 89) with the environment E_4_ (Sylhet) were the most unstable and discriminate while G_2_ (BRRI dhan 28) with mean grain yield (6,406 kg·ha^−1^) was the greater yield-performing genotype in that particular environment. Similar genotypes and environments were represented by related symbols of the IPCA1 score, which suggested a positive association and, as a result, a higher yield of the genotypes in that specific environment. The E_1_ (Dinajpur) environment had positive IPCA1 and IPCA2 scores and registered above average yields and the stable environments for most of the genotypes. While E_4_ (Sylhet) had negative IPCA2 values, thus the Sylhet environment was unfavourable for most of these genotypes.

## 6. Discussion

In the present study, ten Boro rice genotypes underwent evaluation. The partitioning of total variation revealed significant genetic variability and marked differences across the tested traits for all genotypes. Consequently, identifying superior genotypes based on specific morphological attributes positively correlated with grain yield is feasible. Field studies were conducted in geographically and temporally distinct environments, resulting in substantial variance attributed to genotype-environment interaction (GEI). Hence, understanding the sources of variation in genotype performance across different environments was deemed crucial. Akter et al. [24], Sharifi et al. [25], Islam et al. [23], and Kanfany et al. [26] also noted that significant variation in GEI implied the presence of multiple mega-environments, where various genotypes excel in yield performance. Given the diverse grain yield performances of different genotypes across environments, mean comparison analysis sufficed for identifying genotype differences [11, 27]. Comparing means and ranges across environments provides insights into environmental suitability for trait expression. However, the E_3_ (Mymensing) environment exhibited inferior mean performance across traits compared to other environments, making it a less favourable option for achieving higher yields. Conversely, E_2_ (Rangpur) and E_4_ (Sylhet) environments showed promising prospects for cultivation yield. The results highlighted the flexibility of all environments in accommodating a range of yield-contributing traits. Notably, E_4_ (Sylhet) exhibited the widest range across most traits, while E_2_ (Rangpur) demonstrated the widest range for four of the yield-contributing traits. Therefore, these two environments were identified as ideal for genotype screening.

Simple measurements of variability, particularly the genotypic and phenotypic coefficients of variation, are commonly utilized for genotype evaluation [28]. These relative values offer insights into the extent of genetic diversity within a population. Consequently, the variance components, including genotypic and phenotypic variances, were computed. The phenotypic variance was notably greater than the genotypic variance, indicating the influence of both genetic factors and environmental conditions on the expression of the trait under scrutiny. Tewachewu et al. [29] and Hasan et al. [28] reported low variance components for panicle length. Upon analyzing the data above, it became evident that genetic parameters of variance were estimated for all traits across each environment. This observation highlighted high values of *σ*_*g*_^2^ and *σ*_*p*_^2^ for plant height, panicle number per plant, leaf area index, filled grains per plant, unfilled grains per plant, and grain yield. Previous studies by Amegan et al. [30] for grain yield, Limbani et al. [31] for filled grains per plant, and Asante et al. [32] for plant height corroborate these findings. Similarly, Islam et al. [23] recorded analogous results for upland rice genotypes.

The genotypes demonstrated their genetic potential as GCV was slightly lower than the PCV, indicating that genetic factors predominantly influenced the observed characteristics over environmental influences. Traits such as tiller number per plant, harvest index, filled grains per plant, and grain yield consistently exhibited high levels of both GCV and PCV. Comparable findings have been reported by Demeke et al. [33] and Nikhitha et al. [34] for tiller number per plant, and by Fathima et al. [35] and Bhargava et al. [36] for filled grains per plant. Conversely, traits such as total grain weight, panicle length per plant, and unfilled grains per plant exhibited low levels of both GCV and PCV, suggesting limited genetic variation and thus rendering them less suitable as selection criteria for yield improvement. Introduction of diversity through methods such as mutation and recombination, followed by selection, may, therefore, be necessary for enhancing traits such as panicle length [33]. Traits such as plant height, panicle number per plant, total dry weight, 1,000-seed weight, and leaf area index displayed moderate levels of both GCV and PCV. These moderate values suggest that the genotypes possess adequate genetic diversity to facilitate improvement through selection. Similar results were also reported by Lakshmi et al. [37], Singh et al. [38], and Pradhan et al. [39].

Heritability serves as a valuable indicator of how traits are inherited from parents to offspring. Assessments of heritability play a crucial role in determining genetic gains and predicting the accuracy of phenotypic value as a breeding value [33]. A higher percentage of heritability indicates greater variability that is amenable to manipulation. This increased flexibility in variability allows for the potential alteration of trait expression through methods such as mass selection and progeny testing. Previous studies have consistently reported high heritability estimates for most of the studied traits. Ray et al. [40] documented high heritability for panicle length per plant and plant height, while Bhargava et al. [36] and Fathima et al. [35] reported high heritability for 1,000-seed weight, tiller number per plant, and filled grains per plant. Similarly, Nikhitha et al. [34] and Singh et al. [38] found high heritability for tillers per plant, filled grains per plant, and grain yield. Notably, panicle length exhibited high heritability (69.49%) and moderate GAM (12.88%), consistent with findings reported by Demeke et al. [33], Bhargava et al. [36], and Singh et al. [38].

Genetic advance serves as a pivotal indicator of the potential development achievable through the selection of a suitable population [23]. High GAM results signify the capacity for genetic improvement through selection and the maximum control exerted over traits via additive gene action. Consistent findings have been reported by Akabari et al. [41], Abebe et al. [42], Kumari et al. [43], Tripathi et al. [44], and Ray et al. [40]. Across various parameters examined in this research, such as tiller number per plant, 1,000-seed weight, filled grains per plant, unfilled grains per plant, and grain yield, both high heritability and high GAM were observed. This indicates that nonadditive gene effects have minimal influence on these traits, while the influence of additive genes remains strong. Consequently, these attributes may be readily improved through simple selection processes. These findings corroborate the strong heritability and high genetic advance as a percentage of the mean observed for grain yield, as reported by Lakshmi et al. [37]. In contrast, moderately high heritability and moderate GAM were observed for plant height and panicle length. Similar results have been documented in rice by Nikhitha et al. [34], Bhargava et al. [36], and Singh et al. [38]. Low heritability coupled with low genetic advance as a percentage of the mean were noted for leaf area index and total grain weight. The *b*_*i*_ value, being less susceptible to environmental changes, demonstrates greater adaptability when its value is substantially lower. However, if the *b*_*i*_ value is negative, the genotype may only thrive in poor environments.

The first two IPCAs in the AMMI analysis indicated a significant association between the studied attributes and the model's capability to describe stability [21, 45]. Consequently, biplots generated by combining genotypic and environmental scores of the AMMI 1 components provide breeders with a comprehensive understanding of how genotypes, environments, and their interaction (G x E) behave. This study demonstrated that genotype-environment interaction (GEI) significantly contributed to rice yield variance, with AMMI biplots effectively illustrating how genotypes responded to different environments [46]. The key findings, based on AMMI analysis and yield stability, indicated that four of the tested genotypes (G_5_, G_6_, G_7_, and G_8_) performed best in environments E_1_ (Dinajpur) and E_2_ (Rangpur). Consequently, these genotypes should be considered for the Boro rice production area and as a parental base for breeding programs in Bangladesh.

## 7. Conclusions

To identify high-yielding Boro rice genotypes adaptable to diverse regions in Bangladesh, the AMMI statistical model proves invaluable. Our study revealed that genotype G_6_ (BRRI dhan 68) achieved the highest mean yield (6,754 kg ha^−1^), contrasting with G_9_ (BRRI dhan 88) as the lowest (5,620 kg ha^−1^). Notably, G_8_ (BRRI dhan 84), G_7_ (BRRI dhan 81), and G_5_ (BRRI dhan 67) consistently exhibited top grain yields. Environment E2 (Rangpur) displayed exceptional stability, yielding 7,206 kg·ha^−1^ on average. Most genotypes exhibited specificity to certain environments, as depicted in biplots showcasing genotype-environment interaction. The AMMI model categorized environments into four sections, highlighting optimal choices—G_5_, G_6_, G_7_, and G_8_—for areas such as Dinajpur (E_1_) and Rangpur (E_2_). These genotypes hold promise for Boro rice production and future breeding programs in Bangladesh. For future research, additional variables such as fertilizer application, plant diseases, and management systems should be considered.

## Figures and Tables

**Table 1:** Different environments with soil physiological properties.

Parameters	Environment			
	E_1_	E_2_	E_3_	E_4_
Location	Dinajpur	Rangpur	Mymensing	Sylhet
Region	North	North	East	Northeast
Soil texture	Sandy loam	Sandy loam	Silty loam to silty clay	Silty loamy soils
Soil type	Acidic	Acidic	Acidic	Acidic
Soil pH	6.5–7.5	5.5–6.5	5.2–7.8	4.9–6.1

**Table 2:** ANOVA when genotypes were raised in different environments for one year.

Source	d*f*	MSS	Expected MSS
Environment	*s* − 1		
Rep./environment	*s*(*r* − 1)		
Genotype	*v* − 1	MS_1_	*σ*_*e*_^2^+*rσ*_*e*_^2^ + *rsσ*_*g*_^2^
Genotype x environment	(*g* − 1)(*s* − 1)	MS_2_	*σ*_*e*_^2^+*rσ*_*ge*_^2^
Error	*s*(*r* − 1)(*g* − 1)	MS_3_	*σ*_*e*_^2^
Total	*svr*− 1		

Various types of variances (equations (1) and (2)) were performed by the following formula.

**Table 3:** Joint regression analysis of variance.

Sources	d*f*	SS	MSS
Genotype (*v*)	(*v* − 1)	1/s ∑*i*, *y*^2^*i*− C.F.	MS_1_
Environments (*s*)	(*s* − 1)	1/*v*∑*i*, *y*^2^*j*–C.F.	
Genotype × env.	(*v* − 1)(*s* − 1)	∑_*i*_∑_*j*_*Y*_*i*_*j*^2^ − (∑*Y*_*i*_^2^/*s*) − ∑*y*^2^*j*^2^/*v*+CF	
Env. + (var. + Env.)	*v*(*s* − 1)	∑_*i*_∑_*j*_*Y*_*i*_*j*^2^-∑*iY*_*i*_^2^/*s*)	
Env. (linear)	1	1/*v*∑_*i*_(*Y*_*j*_*I*_*j*_)^2^/∑_*j*_*I*_*j*_^2^	
Var. × env. (linear)	*v* − 1	∑*i*/(∑_*i*_*Yi*_*j*_)^2^/∑*I*_*j*_^2^ Env.(linear)ss.	MS_2_
Pooled deviation	*s*(*s* − 2)	∑_*i*_∑_*j*_*σ*_*ij*_^2^	MS_3_
Pooled deviation due to genotype	*v*(*s* − 2)	[∑_*j*_Y_*j*_^2^ − (*Y*_*i*_)^2^/s] − (∑_*j*_*Y*_*ij*_ *ij*)^2^/∑_j_1_j_^2^=∑_*j*_*σ*_*ij*_^2^	
Pooled error	*s*(*r* − 1)(*v* − 1)		MS_4_

*Y*_*ij*_ and *I*_*i*_ are the performance of *i*^th^ genotype at *j*^th^ environment and index, respectively. Mean square deviation from linear regression. *S*_*d*_^2^ is pooled errors as estimated.

**Table 4:** Combined analysis of pooled variance for various morphological traits in ten Boro rice genotypes.

Sources	d*f*	Mean sum of squares					
		1,000-SW	HI	LAI	FG	UFG	GY
Replication (R)	2	46.03^*∗∗*^	6.40^*∗∗*^	0.81^*∗*^	11,2401.00^*∗∗*^	18,191.20^*∗∗*^	9,116,571^*∗∗*^
Genotypes (G)	9	18.20^*∗*^	10.10^*∗∗*^	0.52^*∗∗*^	37,751.00^*∗∗*^	9,082.00^*∗∗*^	1,905,351^*∗∗*^
Environments (E)	3	2,578.14^*∗∗*^	15,229.20^*∗∗*^	0.96^*∗∗*^	19,389.10^*∗∗*^	6,724.50^*∗*^	18,570,558^*∗∗*^
Gen. × env. (G × E)	27	9.20^ns^	11.60^*∗∗*^	0.24^ns^	15,532.00^*∗∗*^	3,775.00^*∗*^	791,247^*∗∗*^
Residuals	78	7.56	1.10	0.17	9,781.00	440.70	670,985
CV		9.55	8.56	22.33	14.05	18.18	13.12
LSD		2.24	0.85	0.34	80.38	37.60	665.76

		PH	TN	PN	PL	TDW	TGW

Replication (R)	2	144.78^*∗∗*^	14.70^*∗∗*^	1.67^ns^	156.11^ns^	80.42^*∗*^	76.57^*∗∗*^
Genotypes (G)	9	89.28^*∗∗*^	6.74^*∗∗*^	7.61^*∗∗*^	22.25^ns^	41.62^*∗*^	18.22^ns^
Environments (E)	3	445.72^*∗∗*^	64.20^*∗∗*^	393.79^*∗∗*^	81.04^*∗∗*^	106.55^*∗∗*^	39.06^*∗∗*^
Gen. × env. (G × E)	27	98.22^*∗∗*^	4.70^*∗∗*^	7.35^*∗∗*^	10.58^ns^	20.94^ns^	315.75^*∗∗*^
Residuals	78	16.39	2.25	2.67	12.68	19.28	9.22
CV		3.40	12.92	11.62	14.09	10.79	13.15
LSD		3.29	1.22	1.33	2.89	3.57	2.47

*Note.* PH = plant height (cm), TN = tiller number (no.), PN = panicle number (no.), PL = panicle length (cm), TDW = total dry weight (g), TGW = total grain weight (g), 1,000-SW = 1,000-seed weight (g), HI = harvest index (%), LAI = leaf area index (%), FG = field grain (no.), UFG = unfilled grain (no.), and GY = grain yield (kg ha^−1^). ns = not significant difference, ^*∗*^ = Significant at *p* < 0.05, ^*∗∗*^ = significant at *p* < 0.01.

**Table 5:** Grain yield of ten Boro rice genotypes among different environments.

Genotypes with code	Yield (kg ha^−1^)				
	Dinajpur (E_1_)	Rangpur (E_2_)	Mymensing (E_3_)	Sylhet (E_4_)	Mean
BRRI dhan 58 (G_1_)	5,535^e^	6,728^d^	5,704^b^	5,234^ab^	5,800
BRRI dhan 28 (G_2_)	5,949^e^	7,013^c^	5,554^b^	6, 204^a^	6,180
BRRI dhan 89 (G_3_)	6,584^c^	6,577^d^	5,238^c^	5,141^ab^	5,885
BRRI dhan 100 (G_4_)	6,426^c^	7,317^b^	5,975^b^	5,644^ab^	6,340
BRRI dhan 67 (G_5_)	6,596^c^	8, 797^a^	6, 123^a^	5,240^ab^	8,405
BRRI dhan 68 (G_6_)	6,149^d^	7,664^b^	5,828^b^	6, 241^a^	6,753
BRRI dhan 81 (G_7_)	7,137^b^	7,657^b^	6, 344^a^	5,685^ab^	6,705
BRRI dhan 84 (G_8_)	7, 744^a^	7,519^b^	6, 302^a^	5,072^b^	6,659
BRRI dhan 88 (G_9_)	5,995^c^	6,038^e^	5,375^c^	5,071^b^	5,619
BRRI dhan 29 (G_10_)	7,228^b^	6,747^d^	5,362^c^	4,953^c^	6,072
Mean	6,534	7,205	5,780	5,448	6,441
LSD (5%)	0.10	0.08	0.11	0.09	
CV (%)	5.36	4.20	5.40	4.03	
*F* test	^*∗∗*^	^*∗∗*^	^*∗∗*^	^*∗∗*^	

*Note.* ^*∗∗*^ = significant at *p* < 0.01. Different superscript letters indicate significant difference within column. Bold values are the highest grain yield from each environments.

**Table 6:** Range, mean variance for yield, and component traits in ten Boro rice genotypes in each environment.

Sl. no.	Characters	Mean				Range			
		E_1_	E_2_	E_3_	E_4_	E_1_	E_2_	E_3_	E_4_
1	PH	113.50	121.50	119.00	**122.00**	96.00–124.00	115.00–124.00	114.00–123.00	117.00–124.00
2	TN	8	12	**13**	10	8–13	11–13	14–16	9–11
3	PN	**17**	9	9	15	15–21	12–15	9–11	14–17
4	PL	**25.67**	24.27	23.31	24.72	24.27-26.20	24.40–29.00	18.00–27.00	22.47-27.63
5	TDW	40.74	38.57	40.26	**43.14**	35.44-43.73	37.13-41.77	36.47-44.53	38.00–45.00
6	TGW	23.26	17.39	22.25	**27.33**	22.00–25.33	14.00–21.00	21.20–23.93	22.95-30.46
7	1,000-SW	24.40	24.00	24.53	**43.00**	22.00–26.67	22-24.33	22.00–28.00	40.00–46.00
8	HI	**45.95**	38.51	41.20	40.20	38.00–50.00	54.00–61.00	45.00–53.00	55.00–62.00
9	LAI	1.68	**2.10**	1.74	1.82	1.46–1.96	1.71–2.39	1.27–2.32	1.49–2.28
10	FG	754	**790**	630	642	579–904	696–904	528–727	557–725
11	UFG	240	254	**275**	248	190–307	189–315	214–368	216–296
12	GY	6,534	**7,205**	5,780	5,448	5,535−7,744	6,038−8,797	5,368−6,344	5,234−6,241

*Note.* PH = plant height (cm), TN = tiller number (no.), PN = panicle number (no.), PL = panicle length (cm), TDW = total dry weight (g), TGW = total grain weight (g), 1,000-SW = 1,000-seed weight (g), HI = harvest index (%), LAI = leaf area index (%), FG = field grain (no.), UFG = unfilled grain (no.), and GY = grain yield (kg ha^−1^). Bold values are the highest mean values among four environments.

**Table 7:** Variance for genotypes and variance for phenotype for yield and component traits in ten Boro rice genotypes in each environment.

Sl. no.	Characters	Variance for genotype (*σ*_*g*_^2^ %)				Variance for phenotype (*σ*_*p*_^2^ %)			
		E_1_	E_2_	E_3_	E_4_	E_1_	E_2_	E_3_	E_4_
1	PH	**14.75**	9.54	14.71	13.40	**23.56**	17.56	17.12	18.20
2	TN	3.77	4.27	**4.71**	3.40	8.45	**8.88**	7.78	6.77
3	PN	**13.99**	12.99	13.16	13.17	**28.72**	23.12	23.11	16.34
4	PL	2.89	4.24	**5.13**	4.67	6.59	8.00	8.40	**8.90**
5	TDW	5.67	10.56	**10.77**	5.71	8.75	8.77	21.23	**9.12**
6	TGW	5.45	**11.86**	5.00	5.10	7.49	**16.23**	6.77	7.89
7	1,000-SW	**6.84**	6.19	6.39	4.03	**13.30**	12.12	12.00	9.30
8	HI	**7.42**	4.11	6.52	4.14	**22.00**	7.67	13.44	9.34
9	LAI	**24.11**	17.25	22.49	24.00	**58.67**	35.00	27.23	28.10
10	FG	11.11	11.13	**12.00**	10.00	21.00	**25.34**	18.34	23.30
11	UFG	11.66	**12.00**	10.35	11.15	21.63	22.13	21.40	**23.44**
12	GY	5,854	6,773	**6,528**	5,554	12,224	12,445	**17,566**	12,456

*Note.* E_1_ = Dinajpur, E_2_ = Rangpur, E_3_ = Mymensing, and E_4_ = Sylhet. Bold values are the highest variance for genotype and phenotype among four environments.

**Table 8:** Genotypic and phenotypic coefficient variance for yield and component traits in ten Boro rice genotypes in each environment.

Sl. no.	Characters	Genotypic coefficient variance (GCV %)				Phenotypic coefficient variance (PCV %)			
		E_1_	E_2_	E_3_	E_4_	E_1_	E_2_	E_3_	E_4_
1	PH	5.10	**8.14**	1.28	2.67	11.89	**14.50**	9.05	8.00
2	TN	13.48	**13.90**	13.55	**13.90**	26.08	26.46	**27.82**	26.46
3	PN	4.61	4.61	**8.35**	4.61	13.12	13.12	15.84	13.12
4	PL	2.23	**2.98**	2.80	2.18	4.27	**4.89**	4.24	4.54
5	TDW	4.32	3.44	1.50	**7.13**	12.98	14.04	12.64	**33.54**
6	TGW	2.02	2.89	**5.66**	3.40	4.34	4.62	5.73	4.12
7	1,000-SW	8.27	8.56	9.81	**10.98**	21.21	25.58	20.30	**28.57**
8	HI	**16.15**	16.09	15.99	**16.15**	**33.88**	**33.88**	23.82	**33.88**
9	LAI	9.77	6.59	8.85	**14.59**	46.36	46.28	46.31	**47.51**
10	FG	**16.90**	15.13	10.00	10.56	17.16	15.47	20.55	**24.33**
11	UFG	2.04	2.04	2.04	2.04	**6.35**	**6.35**	5.31	**6.35**
12	GY	14.23	16.90	13.80	**18.41**	31.77	31.43	**33.26**	30.13

*Note.* E_1_ = Dinajpur, E_2_ = Rangpur, E_3_ = Mymensing, and E_4_ = Sylhet. Bold values are the highest genotypic and phenotypic coefficent variance among four environments.

**Table 9:** Broad sense heritability, genetic advance, genetic advance as a percentage of mean for yield, and component traits in ten Boro rice genotypes in each environment.

Sl. no.	Characters	Broad sense heritability (*H*_*b*_^2^)				Genetic advance (GA %)				Genetic advance as a percentage of mean (GAM %)			
		E_1_	E_2_	E_3_	E_4_	E_1_	E_2_	E_3_	E_4_	E_1_	E_2_	E_3_	E_4_
1	PH	**87.23**	87.11	67.80	33.06	29.86	34.49	**34.54**	30.60	22.39	23.74	**23.81**	22.16
2	TN	86.71	**87.61**	83.72	77.61	**42.12**	41.18	41.16	41.18	34.35	**35.05**	33.59	**35.05**
3	PN	**97.97**	97.37	95.13	74.67	**8.42**	8.22	7.33	8.36	23.82	**32.02**	23.05	23.39
4	PL	62.33	63.30	**83.57**	80.33	11.93	10.93	**12.38**	10.93	13.33	13.33	**19.06**	11.33
5	TDW	61.05	15.99	**61.41**	34.52	**31.16**	30.70	30.14	27.24	**23.96**	21.73	20.37	23.12
6	TGW	85.21	91.21	73.37	**94.78**	21.33	21.24	22.93	**26.57**	6.65	5.90	**9.77**	8.70
7	1,000-SW	80.31	80.31	80.31	**83.31**	**46.35**	42.35	40.35	40.35	**51.35**	**51.35**	34.35	31.35
8	HI	82.73	82.55	**85.06**	82.73	0.07	0.07	**0.10**	0.07	15.87	15.74	22.11	15.87
9	LAI	**24.45**	22.02	13.65	19.42	0.07	0.03	0.05	**0.15**	4.25	1.92	3.48	9.22
10	FG	**96.18**	91.00	93.69	88.82	36.93	27.07	**60.57**	59.29	35.72	37.50	32.03	35.43
11	UFG	**65.10**	61.91	11.31	22.34	32.27	43.48	**68.17**	52.28	63.14	68.72	60.89	63.17
12	GY	**88.40**	81.50	78.99	80.16	32.94	31.33	23.25	**45.72**	46.51	49.40	50.37	52.98

*Note.* E_1_ = Dinajpur, E_2_ = Rangpur, E_3_ = Mymensing, and E_4_ = Sylhet. Bold values are the highest broad sense heritability, genetic advance, and genetic advance as a percentage of mean among four environments.

**Table 10:** Joint regression analysis over different environments of yield characters.

Source	Gen.	Env. + (Gen × Env)	Env. (linear)	Gen × Env (linear)	Pooled deviation	Pooled error
PH	674.00^*∗∗*^	12.20^ns^	8.88	38.20^*∗∗*^	4.14	33.10
TN	545.00^*∗∗*^	14.30^ns^	10.80	40.50^*∗∗*^	4.13	38.90
PN	67.60^*∗∗*^	124.00^ns^	8.82	43.20^*∗∗*^	5.45	41.20
PL	0.56^*∗∗*^	270.00^ns^	5,788.00	5.43^ns^	2.23	0.45
TDW	0.89^*∗∗*^	278.00^ns^	4,455.00	6.43^*∗∗*^	2.24	0.57
TGW	67.90^*∗∗*^	234.00^ns^	12.50	34.50^*∗∗*^	3.44	37.60
1,000-SW	0.88^*∗∗*^	0.09^ns^	1.38	0.07^*∗∗*^	0.006	0.01
HI	66.40^*∗∗*^	292.00^ns^	5,788.00	6.42^*∗*^	1.37	0.35
LAI	45.60^*∗∗*^	230.00^ns^	12.50	33.40^ns^	2.55	0.68
FG	34.50^*∗∗*^	278.00^ns^	15.40	33.50^*∗∗*^	2.67	0.23
UFG	23.50^*∗∗*^	289.00^ns^	23.40	35.70^*∗∗*^	1.89	0.12
GY	2.46^*∗∗*^	1.02^ns^	2.26	0.04^*∗∗*^	0.005	0.003
d*f*	9	30	2	3	15	40

*Note.* ns = not significant difference, ^∗^ = Signifcant at *p* < 0.05, ^*∗∗*^ = signifcant at *p* < 0.01.

**Table 11:** Stability parameters (*b*_*i*_ and *S*_*d*_^2^) of ten Boro rice genotypes for plant height, tiller number per plant, panicle number per plant, panicle length, leaf area index, and harvest index.

Genotypes	PH		TN		PN		PL		LAI		HI	
	*b*_*i*_	*S*_*d*_^2^	*b*_*i*_	*S*_*d*_^2^	*b*_*i*_	*S*_*d*_^2^	*b*_*i*_	*S*_*d*_^2^	*b*_*i*_	*S*_*d*_^2^	*b*_*i*_	*S*_*d*_^2^
BRRI dhan 58 (G_1_)	1.18	0.13	1.42	−0.54	1.04	−0.71	0.20	−7.20	0.76	1.03	1.01	0.06
BRRI dhan 28 (G_2_)	0.97	−0.12	0.12	−0.65	0.93	−1.61	0.22	−10.70	0.97	−0.15	0.92	−0.01
BRRI dhan 89 (G_3_)	−0.39	−0.11	−0.85	−0.75	1.08	−1.56	0.33	−10.40	1.22	0.07	−0.41	0.25
BRRI dhan 100 (G_4_)	0.99	0.08	1.20	−0.07	1.26	−0.82	0.83	−10.10	1.64	0.01	0.92	0.24
BRRI dhan 67 (G_5_)	0.89	−0.08	0.41	−0.70	0.69	−1.10	−0.02	−7.10	0.40	0.05	0.84	0.28
BRRI dhan 68 (G_6_)	1.04	0.005	1.23	−0.54	1.10	0.78	0.45	−5.11	0.78	−0.11	0.97	0.11
BRRI dhan 81 (G_7_)	−0.12	0.002	1.02	−0.56	1.33	−0.87	0.89	4.78	0.89	0.07	0.87	−0.33
BRRI dhan 84 (G_8_)	−0.10	−0.11	1.11	−0.08	1.23	−1.10	1.04	−5.60	0.83	0.05	0.30	−0.23
BRRI dhan 88 (G_9_)	0.89	0.20	−1.03	−0.73	0.97	−1.23	1.03	−3.22	−0.23	0.22	−0.11	0.03
BRRI dhan 29 (G_10_)	0.92	0.34	1.40	−0.55	0.89	−1.02	0.92	−3.02	−0.02	−0.06	−0.33	0.23

Genotypes	TDW		TGW		1,000-SW		FG		UFG		GY	
	*b*_*i*_	*S*_*d*_^2^	*b*_*i*_	*S*_*d*_^2^	*b*_*i*_	*S*_*d*_^2^	*b*_*i*_	*S*_*d*_^2^	*b*_*i*_	*S*_*d*_^2^	*b*_*i*_	*S*_*d*_^2^

BRRI dhan 58 (G_1_)	0.96	−0.34	0.42	−0.54	1.02	0.04	0.20	−7.20	0.76	−0.71	0.16	−0.01
BRRI dhan 28 (G_2_)	0.24	1.47	0.54	−0.65	1.79	−0.02	0.22	−6.70	0.97	0.61	0.87	−0.02
BRRI dhan 89 (G_3_)	1.00	0.02	−0.85	−0.75	1.12	−0.07	0.33	−6.40	1.22	0.34	0.95	0.03
BRRI dhan 100 (G_4_)	1.03	−0.16	0.78	−0.07	0.56	0.05	0.83	−5.10	1.64	−0.58	0.87	0.04
BRRI dhan 67 (G_5_)	0.89	1.47	0.81	−0.70	0.47	−0.17	−0.02	−7.10	0.40	−0.43	0.73	−0.01
BRRI dhan 68 (G_6_)	1.04	0.07	0.23	−0.54	1.10	0.78	0.45	−5.11	0.78	0.78	−0.23	0.05
BRRI dhan 81 (G_7_)	−0.12	0.05	1.02	−0.56	1.33	−0.87	0.89	4.78	0.89	−0.34	0.24	0.23
BRRI dhan 84 (G_8_)	−0.10	−0.11	−0.11	−0.08	1.23	−1.10	1.04	−5.60	0.83	−0.45	0.98	−0.05
BRRI dhan 88 (G_9_)	0.87	0.22	0.83	−0.73	0.97	−1.23	1.03	−3.22	0.23	0.89	0.22	−3.11
BRRI dhan 29 (G_10_)	0.90	0.32	0.40	−0.55	0.89	−1.02	0.92	−3.02	0.02	−0.11	0.89	−1.22

**Table 12:** AMMI analysis of variance including the partitioning of GEI of grain yield.

Sources	d*f*	SS	MS	TSS explained (%)
Environment (E)	3	55,711,675	18,570,558.30	10.23^*∗∗*^
Genotype (G)	9	17,148,157	1,905,350.80	80.60^*∗∗*^
Interaction (G × E)	27	21,363,671	791,247.10	9.17^*∗∗*^
AMMI component 1	11	14,216,505	1,292,409.50	66.50^*∗∗*^
AMMI component 2	9	5,069,635	563,292.80	23.70^*∗∗*^
AMMI component 3	7	2,077,530	296,790.00	9.80
Residuals	72	40,852,159		
Total	146	186,157,137		

*Note.* ^*∗∗*^ = signifcant at *p* < 0.01.

**Table 13:** AMMI analysis showing means with IPCA1, IPCA2, and IPCA3 scores of grain yield for ten Boro rice genotypes grown in four environments.

Genotypes with code	Mean	Grain yield (kg ha^−1^)		
		PCA1	PCA2	PCA3
BRRI dhan 58 (G_1_)	5,801	−11.0	7.0	15.0
BRRI dhan 28 (G_2_)	6,406	−0.1	12.0	11.0
BRRI dhan 89 (G_3_)	6,341	8.3	5.3	−10.0
BRRI dhan 100 (G_4_)	5,886	−4.4	2.1	4.1
BRRI dhan 67 (G_5_)	6,073	20.0	−1.5	−13.0
BRRI dhan 68 (G_6_)	6,754	28.0	20.0	−9.3
BRRI dhan 81 (G_7_)	6,705	4.9	−3.3	4.2
BRRI dhan 84 (G_8_)	6,660	22.0	−12.0	3.9
BRRI dhan 88 (G_9_)	5,620	3.9	17.0	3.7
BRRI dhan 29 (G_10_)	6,180	−16.0	16.0	−8.6
E_1_ (Dinajpur)	6,535	37.0	−1.3	−11.0
E_2_ (Rangpur)	7,206	−18.0	−28.0	−2.6
E_3_ (Mymensing)	5,781	3.9	6.6	24.0
E_4_ (Sylhet)	5,449	−22.0	−22.0	−11.0

## Data Availability

All data used to support the findings of this study are available from the corresponding author upon request.
